# Analysis of the Propionate Metabolism in *Bacillus subtilis* during 3-Indolacetic Production

**DOI:** 10.3390/microorganisms10122352

**Published:** 2022-11-28

**Authors:** Freddy Castillo-Alfonso, Alejandro Quintana-Menéndez, Gabriel Vigueras-Ramírez, Alfonso Mauricio Sales-Cruz, Luis Manuel Rosales-Colunga, Roberto Olivares-Hernández

**Affiliations:** 1 Posgrado en Ciencias Naturales e Ingeniería, Universidad Autónoma Metropolitana Unidad Cuajimalpa, Ciudad de México 05370, Mexico; 2 Departamento de Procesos y Tecnología, Universidad Autónoma Metropolitana, Unidad Cuajimalpa, Av. Vasco de Quiroga 4871, Col. Santa Fe Cuajimalpa, Cuajimalpa de Morelos, Ciudad de México 05348, Mexico; 3 Facultad de Ingeniería, Universidad Autónoma de San Luis Potosí, Av. Dr Manuel Nava 8, Zona Universitaria, San Luis Potosí 78290, Mexico

**Keywords:** *Bacillus subtilis*, propionate metabolism, 3-indole acetic acid, flux balance analysis

## Abstract

The genera *Bacillus* belongs to the group of microorganisms that are known as plant growth-promoting bacteria, their metabolism has evolved to produce molecules that benefit the growth of the plant, and the production of 3-indole acetic acid (IAA) is part of its secondary metabolism. In this work, *Bacillus subtilis* was cultivated in a bioreactor to produce IAA using propionate and glucose as carbon sources in an M9-modified media; in both cases, tryptophan was added as a co-substrate. The yield of IAA using propionate is 17% higher compared to glucose. After 48 h of cultivation, the final concentration was 310 mg IAA/L using propionate and 230 mg IAA/L using glucose, with a concentration of 500 mg Trp/L. To gain more insight into propionate metabolism and its advantages, the genome-scale metabolic model of *B. subtilis* (*i*BSU 1147) and computational analysis were used to calculate flux distribution and evaluate the metabolic capabilities to produce IAA using propionate. The metabolic fluxes demonstrate that propionate uptake favors the production of precursors needed for the synthesis of the hormone, and the sensitivity analysis shows that the control of a specific growth rate has a positive impact on the production of IAA.

## 1. Introduction

3-Indoleacetic acid (IAA) is a carboxylic acid that belongs to the group of phytohormones called auxins, which has a role as a plant growth hormone [1]. It is responsible for increasing the density of root hairs and lateral roots favoring the consumption of nutrients from the surroundings. At the cellular level, it stimulates the proliferation of cells in the young areas of the plant and contributes to the differentiation of vascular tissues [2]. These types of hormones are produced by the so-called plant growth-promoting bacteria (PGPB), which are part of the genera: *Azotobacter* sp., *Azospirillum* sp., *Pseudomonas* sp., *Bacillus* sp. The increasing demand for sustainable handling in agriculture activity, stands out the potential of the PGPB to produce biofertilizers that have an impact on the yield and quality of commercial farming [3]. The synthesis of IAA is considered part of the secondary metabolism of *B.subtilis,* and the synthesis takes place during the transition from the exponential phase to the stationary phase of bacterial growth. The main precursor in the synthesis is the amino acid L-tryptophan (Trp) as it provides the carbon skeleton of this phytohormone. In general, there are several routes of synthesis of IAA in the group of PGPB: the indole 3-pyruvate (IPA), the tryptamine (TAM) and the indole 3-acetamide (IAM).

From the group of the PGPB, *B. subtilis* has been widely used for industrial development to produce different compounds of high value such as enzymes, antibiotics, nucleotides and polyhydroxybutyrate (PHB). One of the main bases for using *B. subtilis* in these biotechnological applications is the knowledge of its central carbon metabolism and its adaptation to different environments. Traditionally, the production processes are carried out using mainly glucose or other sugars as a carbon source [4,5], nevertheless, it has the ability to grow on citrate, glycerol, mannitol and L-glutamic acid as a carbon source [6,7]. It also can grow in different organic acids such as acetate, propionate, butyrate, succinate and formate [8]. Depending on the point of entry of the carbon source in the metabolic pathway, the efficiency to produce metabolic intermediates varies considerably [9].

Initial studies on IAA production using *B. subtilis,* isolated from the rhizosphere in soy plants in Brazil, reported the concentration from 170 mg IAA/L to 310 mg IAA/L using a complex media with potato-dextrose, hydrolyzed protein and glucose as the main carbon source [10]. Another study reported the production of 55 mg IAA/L cultivating *B. subtilis* SJ-101 in M9 minimal media with 500 mg Trp/L [11]. The isolation of *Bacillus velezensis* FKM10 from the rhizosphere in apple trees was used to demonstrate its capability to promote plant growth [12] and it can make synergy with other microorganisms from the genera *Aspergillus* [13]. Alternatively, the sole production of IAA in other microorganisms are reported. Based on the strategy of culture conditions optimization, *Streptomyces fradiae* NKZ-259 was cultivated to overproduce IAA reaching a four-fold increase (82.363 μg/mL) with 2 g Trp/L. The cell culture and the culture supernatant filtrated were tested in *Solanum lycopersicum* plants comparing root length and dry and fresh plant weight reporting a 20% increase on average in those plants subject to the microorganism as promoter [14]. The well-known industrial microorganism *Escherichia coli* was genetically engineered to overproduce IAA introducing the de novo pathway IAM. The strain was capable to produce 906 mg IAA/L using a complex media [15]. The large-scale production was also exploring cultivating *Rhodosporidiobolus fluvialis* DMKU-CP293 strain in a pilot-scale bioreactor with a volume of 100 L. The final titer was 3569.32 mg IAA/L, which is so far, the first report of IAA production at the pilot scale [16]. The cultivation media, in the cases mentioned above, was a complex media and Trp dependent, despite the high titer, the production cost can be reduced if minimal media can be used together with the advantages of a natural producer such as *B. subtilis*. 

The cultivation strategies to over-produce the desired metabolite can be directed with rational approaches, such as the mathematical modeling of the metabolism and computational tools. For instance, genome-scale metabolic models (GSMM) compile gene-protein-reaction associations of the metabolic network at genome wide level [17]. Based on this information, it is possible to build a mathematical framework that includes optimization algorithms for flux predictions, known as constrain-based modeling. In particular, the framework for flux predictions using linear programming is called flux balance analysis (FBA) [18,19]. This approach has been widely used for the exploration of metabolic engineering strategies, such as gene deletion, gene insertions, production yields or substrate utilization [20]. As with other mathematical frameworks, FBA has some limitations, one of these is the multiple solutions for flux distribution and competition among metabolic objectives. To overcome these problems, other constraint-based algorithms have been developed. For example, the overproduction of desired metabolites decreases biomass formation, to simulate this scenario, a bilevel optimization framework was developed with the purpose to find gene deletion strategies to maximize biomass formation with maximization of the production of the desired metabolite [21]. 

Recently, by using the GSMM of *Bacillus* and constraint-based approaches it was possible to predict the overexpression of target genes to increase the production of fengycin, a novel antifungal [22]. The constraint-based analysis of the metabolic network includes tools to evaluate the robustness and phenotypic states to achieve the desired production objectives [23,24]. Robustness analysis is a constraint-based analysis to assess how much an objective function will change as a function of change in a particular reaction flux [25,26,27]. Another method is the single gene deletion analysis, this is used to potentially find gene deletions to overproduce or inhibit compounds trying to preserve the same distribution of fluxes. Finding the optimal phenotype by eliminating components of the metabolic network is one of the goals of using these tools [28]. The analysis of the Phenotype Phase Plane (PhPP) identifies the dependencies on environmental conditions to optimize target fluxes [29].

In this work, we explored the production of IAA cultivation of *B. subtilis* in minimal media M9 and an alternative carbon source such as propionate. GSMM allowed the exploration of the metabolic pathway activity highlighting key aspects of the propionate distribution and its impact in IAA production. Additionally, constraint-based analysis addresses the need to control the growth rate in order to increase the IAA productivity. 

## 2. Materials and Methods

### 2.1. Strain and Media Cultivation

The strain *Bacillus subtilis* W168 was taken from the cellular bank from Laboratorio de Biotecnología, UAM Cuajimalpa. This strain is prototrophic and sporulating. For the cultivation a minimal media M9 was adjusted based on the Carbon/Nitrogen ratio with a composition of 6 g Na_2_HPO_4_/L, 3 g K_2_HPO_4_/L, 0.5 g NaCl/L, 0.5 g NH_4_NO_3_/L, 0.024 g MgSO_4_/L, 0.0001 g CaCl/L. The carbon sources were 5 g/L of propionate and glucose and a concentration of 0.5 g/L of tryptophan as a co-substrate.

### 2.2. Bioreactor Cultivation

The inoculum was prepared and incubated at 33 °C for 24 h, and seed into the bioreactor New Brunswick BioFlo III with 3 L capacity (working volume of 2 L) The bioreactor is equipped with a sensor for pH, dissolved oxygen (DO) and temperature. The gases, O_2_ and CO_2_ were monitored using the Blue Sense during the time of cultivation. The cultivation time was 48 h and stirred at 250 rpm. The samples were taken at 0, 4, 6, 15, 16, 17, 18, 19, 21, 23, 25, 40, 44 and 48 h, with a volume of 2 mL. The samples were centrifuged at 12,000 rpm for 5 min. The supernatant was used to quantify the concentration of glucose, propionate, Trp and IAA, and the pellet to quantify biomass. Before scaling up to the bioreactor, flask cultivations were evaluated to identify the variability on the experimental data (See supplementary material 1) with technical and biological replicates. Based on these results and the small standard deviation observed at flask level, we took the decision to perform one experiment at the bioreactor level as the variation might be at the same level. The same level of variation was reported previously [30]. 

### 2.3. Biomass and Metabolite Quantification

The biomass pellets were dried at 80 °C for 24 h and the weight was registered. The absorbance at 600 nm with a spectrophotometer (BioPho-tometer plus, Eppendorf, Framingham, MA, USA) for each sample was taken to prepare the calibration curve. Carbon sources were quantified by HPLC (ProStar, Varian, Palo Alto, CA, USA) with an Aminex HPX-87H column (Bio-Rad Laboratories Inc., Hercules, CA, USA) equipped with a refraction index detector (Smart Line 2300, Knauer, Berlin, Germany). The mobile phase was sulfuric acid (H_2_SO_4_) at 5 mM. The flow of the mobile phase was 0.6 mL/min. 

IAA and tryptophan quantification was determined with spectrophotometry at a wavelength of 530 nm (BioPho-tometer plus, Eppendorf, Framingham, MA, USA) applying the Salkowski method [31]. The samples with the supernatant were incubated for 30 min in dark conditions with the Salkowski reactive (2.4 g de FeCl_3_ in 100 mL de H_2_SO_4_) with a ratio of 1:1. To obtain the values of IAA the absorbance values were extrapolated using a calibration curve obtained with synthetic IAA (Sigma and Aldrich, St. Louis, MO, USA). These values were corroborated with HPLC. The samples were defrosted and filtered with nylon filters with a particle size of 0.2 µm. The analysis was performed on a ProStar HPLC system (Varian, Palo Alto, CA, USA) using a C18 Spherisorb ODS2 column (5 µm, 150 × 4.6 mm ID; Waters, Milford, MA, USA), equipped with a UV–Vis detector adjusted at 280 nm. The mobile phase was acidulated water (acetic acid at 1% *v*/*v*) and acetonitrile in a ratio of 70:30. The flow was 1 mL/min. The calibration curve for IAA and tryptophan was prepared using a range of concentration 0.001 to 1 g/L.

### 2.4. Genome-Scale Metabolic Model and Flux Calculations

The genome-scale metabolic model used for the flux calculations was the *i*BSU 1147 + 1 [30], this model includes a missing reaction from the previous version, the *i*BSU 1147 [32]. The model accounts for 1148 genes and 1745 reactions, these reactions are distributed in 1199 metabolic reactions, 290 for transport and 255 for exchange, and 1457 metabolites divided into 1202 intracellular and 255 extracellular [25,33].

The fluxes were calculated using flux balance analysis (FBA). This methodology is based on the linear programming algorithm where the objective function is the biomass, as the initial objective, and the restrictions are a set of linear equations accounting for the mass balances of each internal metabolite. Other restrictions are applied such as the specific production and consumption rates calculated from the kinetic cultivation data. 

The mathematical formulation of the FBA problem is formulated with the following equation:

$$max/min\;Z=v_{biomass}$$

$$st.\;\;\;\;\;S\cdot \vec{v}=0$$

$$v_{j}=v_{ex,j}$$

(1) $$\alpha \leq \;\vec{v}\leq \beta \;\;\;\;\;\;\;\;\alpha ,\beta \;\epsilon \;\mathbb{R}\;$$

where S is the matrix where the entries are the stoichiometric coefficients of the metabolites participating in the metabolic reaction. The vector $\vec{v}$ is the flux of each metabolic reaction in the model. The objective function Z is the flux (specific growth rate) in the pseudo-biomass reaction, but other physiological objectives can be represented [18,34]. The variable $v_{ex,j}$ are the specific consumption and production rates; in this work, the specific production rate of IAA and the specific consumption rates of glucose, propionate and tryptophan were calculated based on the kinetic cultivation data, and these are used as restrictions for the optimization problem. When the objective function was the production of IAA, the specific growth rate was restricted together with the carbon source rates. 

### 2.5. Constrain-Based Analyses: Robustness, Gene Deletion, Phenotypic Phase Plane and OptKnock

The sensitivity analysis in linear programing is performed by evaluating the changes in the objective function as a function of the changes in the decision variables or modifications in the restrictions of interest. In the context of FBA, the reduced cost evaluates the changes in the objective function relative to the changes in the flux of the reaction of interest. The shadow price evaluates the changes in the objective function relative to an increase in a particular metabolite [35]. These approaches for the sensitivity analysis in linear programming are the bases for the single and doubles robustness analysis and the phenotypic phase plane. The robustness analysis considers the variations in the enzyme activity by changing the flux value in a particular reaction and how these change the impact in IAA production. The phenotypic phase plane evaluates the availability of two metabolites, oxygen and IAA, and their impact in growth rate. Single-gene deletion analysis allows the search of all possible mutants that increase IAA production. The computational analyses were carried out with the Cobra Toolbox 3.0 [29] in Matlab 2021^®^. The evaluation of two objectives we performed using a bilevel optimization algorithm using OptKnock [36]. This bilevel algorithm enables the search of two objectives: the maximization of the growth cell imposed by the maximum IAA production, the files with the Matlab codes and GSMM of *B. subtilis* are in Supplementary material 3–7.

### 2.6. Statistical Analysis and Data Chart

The statistical test to evaluate the kinetics parameters was the Kruskal–Wallis test, this test uses the χ^2^ statistic for no normal distributed data and based on this statistical analysis we found no significant differences across replicates in flask cultivations and HPLC data (See supplementary material 1) [37]. All data presented were filtered and analyzed using the Microsoft Excel statistical package [38]. All graphs presented in the manuscript were built using Matlab (Mathworks, Inc., Natick, MA, USA). 

The specific growth rate, *µ* (h^−1^), was calculated using a linear regression of the biomass concentration vs. IVC (Area under the curve using the Simpson numerical method). The specific rate of substrate consumption, *q_s_*, was calculated with a linear regression of the substrate concentration vs. IVC, the *q_p_* was calculated with a linear regression of the product concentration vs. IVC, and the concentrations of biomass, substrate and product used were in mmol/L units and the IVC value was in gDW h/L units. *Y_x_*_/s_, *Y*_x/p_ and *Y*_p/s_ yields were determined from the kinetic parameters *µ*, *q_s_*, *q_p_*.

## 3. Results

### 3.1. Bioreactor Cultivations

The product and biomass yields are dependent on the carbon source and culture conditions; in particular, the metabolism of *B. subtilis* produces IAA requiring tryptophan (Trp) as a precursor. Considering this Trp dependency, after the evaluation of IAA production in shake flask cultivations comparing five different carbon sources, it was possible to identify the propionate as an optimal carbon source to increase product yield [30]. To have more insights into propionate’s metabolism, we report the kinetic parameters from microbial cultivations in 3 L bioreactor using propionate and glucose as a reference.

#### 3.1.1. Cultivation Kinetics

**Figure 1:**
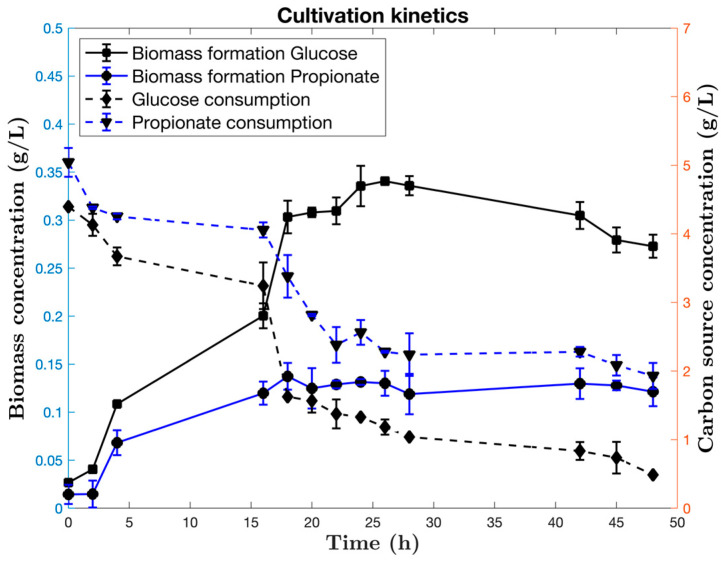
Cultivation kinetics of *B. subtilis* using glucose and propionate as carbon sources. Minimal media M9 was used, and tryptophan added at 16 h.

*Bacillus subtilis* was cultivated in a 3 L bioreactor with air, temperature and pH control. Tryptophan was added as precursor for the IAA production, the biomass production and glucose and propionate consumptions can be seen in Figure 1.

The cultivation time was extended up to 48 h. The initiation of the stationary growth phase is close to 18 h; therefore, the induction with Trp started at 16 h. The stationary phase is sustained up to 42 h of cultivation, after that, the decline phase is observed. The final biomass concentration was 0.34 g /L on glucose compared to the 0.13 g DW/L when propionate is the carbon source. These values are reached close to 18 h and kept almost invariant during the stationary phase. Glucose is almost consumed, whereas 1.92 g/L of propionate remains at 48 h. The same tendency was reported in shake flask cultivations [30].

#### 3.1.2. IAA Kinetics

**Figure 2:**
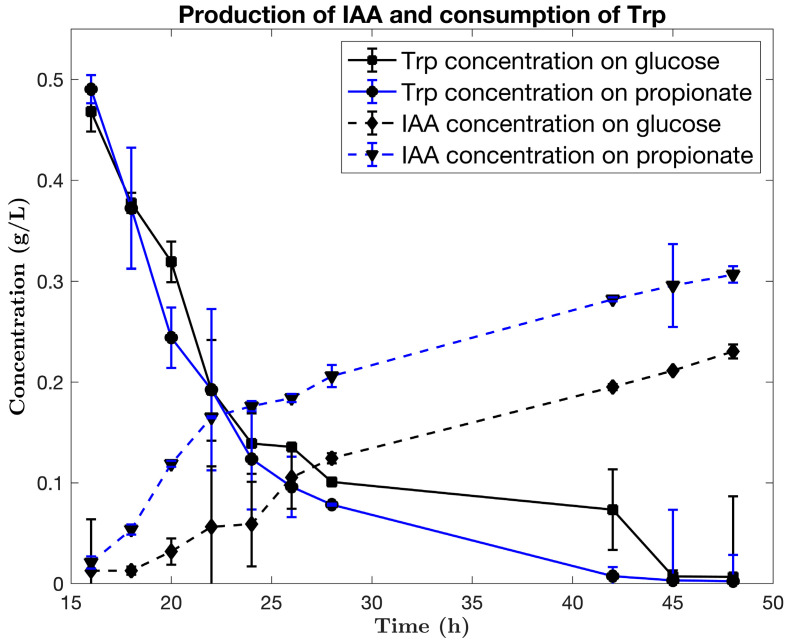
Kinetics of tryptophan consumption and IAA production using glucose and propionate as carbon source.

In Figure 2, it can be seen the kinetics of IAA production coupled with the Trp consumption when comparing glucose and propionate as carbon sources. Trp induction was made at 16 h after the initiation of the cultivation. 

The accumulation of the product is extended up to 48 h, as the metabolism of *B. subtills* might be active while the Trp is present in the media. The final concentration of AIA is 0.30 g/L growing on propionate, compared to 0.23 g/L growing on glucose.

#### 3.1.3. Kinetic Parameters

The kinetic parameters are presented in Table 1. In terms of biomass formation, glucose presented a higher yield (Y_x/s_ = 0.324 ± 0.05) compared to propionate (Y_x/s_ = 0.219 ± 0.08). The most interesting observation is that IAA yield (Y_p/(s+Trp)_ = 0.524 ± 0.04) is higher when propionate is the carbon source. The same situation can be seen with productivity, it is higher with propionate (*q*_p,IAA_ = 1.92 ± 0.07) compared to glucose (*q*_p,IAA_ = 1.33 ± 0.02).

### 3.2. Flux Distributions

**Figure 3:**
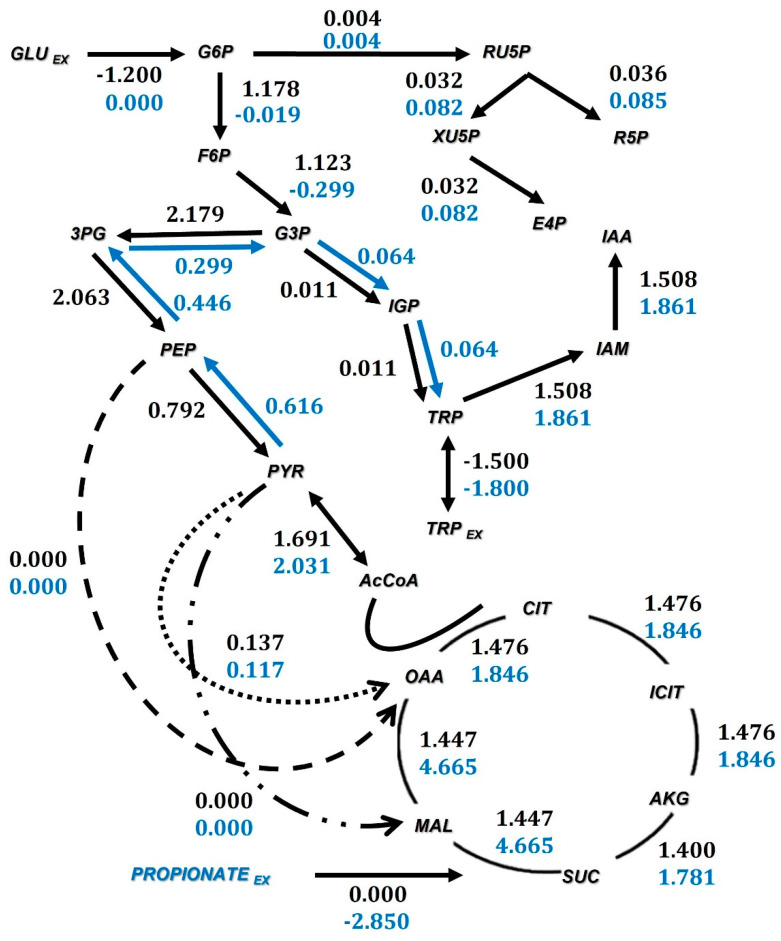
Flux distribution in the central carbon metabolism. Numbers in black are the fluxes when glucose is the carbon source and blue numbers when propionate is the carbon source. These fluxes are calculated in the stationary growth phase with units of mmol/(gDW h). The set of reactions are annotated in Appendix A.

**Figure 4:**
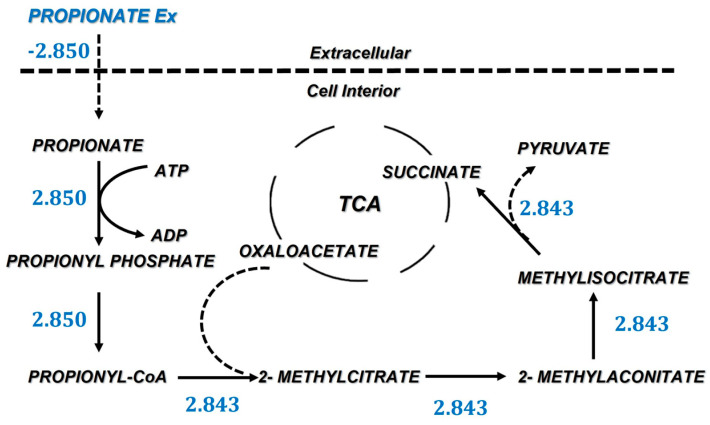
Metabolic pathway and fluxes for the incorporation of propionate to central carbon metabolism.

The kinetic parameters reported in Table 1 were used to calculate the flux distributions (*q*_s,stat_, *q*_s,Trp_, *μ*_stat_, and *q*_p,IAA_). In Figure 3, the flux distribution is mapped across the central carbon metabolism when propionate and glucose are the carbon sources used. The incorporation of propionate into the *B. subtilis* catabolism occurs through the methylcitric acid pathway; an intermediate in the synthesis of fatty acids and the tricarboxylic acid cycle [39]. Glucose enters through the glycolytic pathway. When propionate is the carbon source, there is an increase in the flux value at the level of succinate to form malate that corresponds to the incorporation of this carbon source to the TCA cycle (see Figure 4). The increase in the flux value in the formation of succinate is due to the synergistic effect in the formation of this metabolite via alpha-ketoglutarate and 2-methylcitrate. *Bacillus subtilis* has the anaplerotic pathway responsible for forming phosphoenolpyruvate from oxalacetate by the enzyme phosphoenolpyruvate carboxykinase (PEPCK) and forming pyruvate from oxalacetate by the enzyme pyruvate kinase (PYR). It also possesses the ability to form pyruvate from malate through the malic enzymes encoded in the *sfcA* and *maeA* genes. IAA production using propionate can activate or modulate these anaplerotic pathways. The pentose phosphate pathway (PPP) allows the generation of NADPH, but this product can be supplied by tricarboxylic acids cycle (TCA) activity. When TCA activity is decreased, this metabolic pathway can be activated to generate a number of intermediates, as can be seen when analyzing the flux values in the PPP. Furthermore, the formation of glyceraldehyde 3-phosphate from erythrose 4 -phosphate is favored to form tryptophan via indolglycerol phosphate as the main synthesis pathway of this amino acid from erythrose 4-phosphate via shikimate and corismate is found repressed by the Trp operon [40].

The main pathway for the synthesis of Trp is through the molecules of glyceraldehyde 3-phosphate, erythrose 4-phosphate, phosphoenolpyruvate, shikimate and chorismate; all intermediaries of the formation of Trp, and consequently, of IAA. Therefore, analyzing the behavior of the generation and consumption of these compounds, mainly glyceraldehyde 3-phosphate and erythrose 4-phosphate, can be indicative of how intracellular variations of these substances impact the production of IAA by participating in the formation of indole intermediaries.

### 3.3. Propionate Uptake Pathway

Propionate is incorporated into the metabolism at the TCA level, specifically through succinate. In Figure 4, the incorporation requires six enzymatic steps. Oxaloacetate is incorporated to produce 2-methylcitrate in an irreversible step using the 2-methylcitrate synthase (EC 2.3.3.5). Oxaloacetate is an intermediary in the TCA, therefore the activity of the TCA increases to provide this intermediate as a reactive in the reactions that consume propionate, and this carbon is recycled back to succinate but also into pyruvate, as in the final step methylisocitrate is converted to succinate and pyruvate using the enzyme methylisocitrate lyase (EC 4.1.3.30); further, the pyruvate (PYR) is converted to phosphoenolpyruvate (PEP) in the reaction catalyzed by the phosphoenolpyruvate synthase (EC 2.7.9.2) replenishing the gluconeogenesis activity. 

### 3.4. Analysis of Metabolic Capabilities

#### 3.4.1. Simple Robustness Analysis

**Figure 5:**
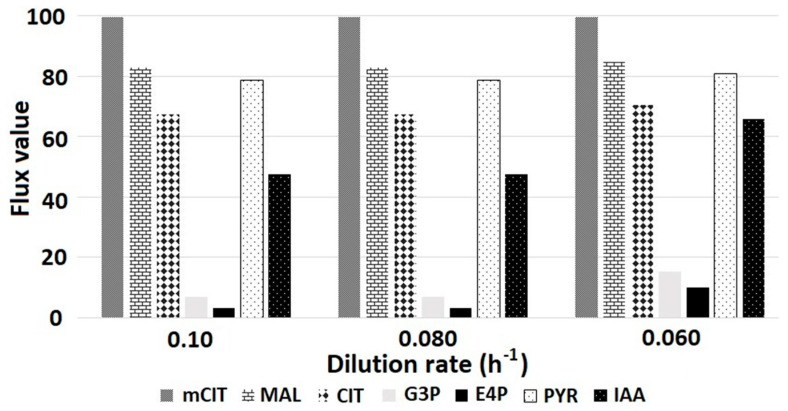
Normalized fluxes as a function of specific growth rate, in this case, represented as dilution rate. The consumption rates of sodium propionate (*q*_s_) are 3.5, 3.0 and 2.5 mmol/L corresponding to the specific growth rates m of 0:06, 0:08 and 0:10 h^−1^, respectively. Fluxes were normalized based on the propionate consumption rates and represented as percentage.

The presence of the precursor for IAA production is evaluated at different growth rates. Seven reactions that participate in the formation of the following products were selected: 2-methylcitrate (mCIT), malate (MAL), citrate (CIT), glyceraldehyde 3-phosphate (G3P), erythrose 4-phosphate (E4P), pyruvate (PYR) and IAA. This set of metabolites is involved in propionate metabolism and IAA production according to the flux distributions evaluated previously. The different flux values for these reactions were obtained by simulating different values of the specific growth rates (*μ*); in terms of cultivation strategies of *Bacillus subtilis,* this analysis suggests it is similar to performing experiments with different dilution rates in chemostat cultivation. All the values of metabolic fluxes obtained were normalized considering the specific consumption rate of propionate, in order to obtain independent data from the initial concentration and to be able to withdraw conclusions of the behavior of the network depending only on the biomass values. As can be seen in Figure 5, the values of glyceraldehyde 3-phosphate (G3P), erythrose 4-phosphate (E4P) and IAA increase as the biomass (m) decreases while simulating the optimal IAA production. Glyceraldehyde 3-phosphate (G3P) and erythrose 4-phosphate (E4P) are primary metabolic precursors for the synthesis of this phytohormone; therefore, their intracellular concentration must increase as the metabolism is diverting resources towards obtaining these precursors [15].

**Figure 6:**
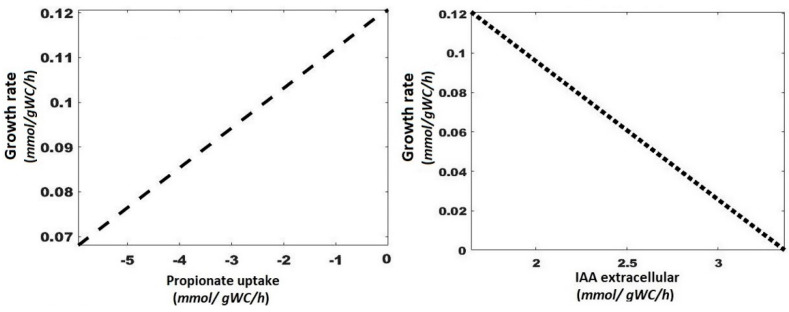
Simple robustness analysis of IAA formation metabolism and propionate consumption as a function of growth rate. Negative values of the propionate uptake are indicating that this substrate is consumed.

Figure 6 shows the computational analysis of the variation of the flux through a reaction of interest and the optimal value of the objective function calculated for each variation, allowing to evaluate how sensitive the objective function is to this particular reaction. The analysis predicts the changes and directionality of the metabolism; as it predicts how the biomass concentration should vary in the formation of a specific metabolite, in this case, an increase in biomass impacts the formation of IAA. From the predicted values, the formation of IAA increases as the growth rate decreases, improving the consumption of Trp, diverting the incorporation of this amino acid to essential proteins and being free for synthesis as it begins to produce IAA in a state where biomass is still being formed.

#### 3.4.2. Double Robustness Analysis

**Figure 7:**
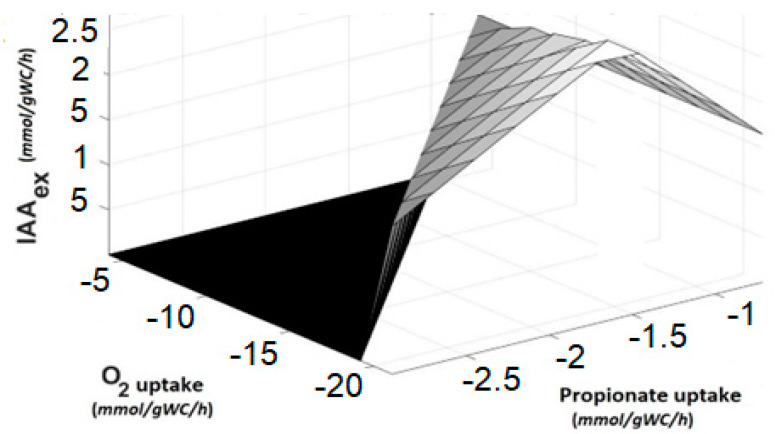
Double robustness analysis of IAA production rate as a function of propionate uptake and O_2_ consumption. Negative values of the propionate and oxygen uptake are indicating that these substrates are consumed.

To evaluate the changes in the oxygen and propionate uptake in the IAA production, the double robustness analysis is presented in Figure 7. The surface graph presents the flux towards IAA as a function of oxygen and propionate uptakes (negative values), the maximum flux value of IAA is predicted when oxygen uptake has the maximum value, but not for propionate uptake. For this simulation, the Trp uptake flux was fixed, and the excess of propionate does not imply an increment in IAA. On the other hand, the increase in O_2_ availability increases the activity of the enzyme Trp 2-monooxygenase allowing a greater synthesis of IAA.

#### 3.4.3. Single Gene Deletion

A total of 271 essential genes were reported experimentally for *B. subtilis* by Kobayashu et al. [41] and 257 by Koo et al. [42] when it was cultivated in the LB medium at 37 °C. In the gene deletion analysis during the simulation of growth in propionate to overproduce IAA, we identified 249 essential genes (See supplementary material 8). The IAA production during the simulation with the model iBSU 1147 + 1 using propionate and Trp was 1.71 mmol/(gDW h), converting the 97.5 % of the Trp flux considered in the simulations. As can be seen in Appendix B, when applying the gene elimination strategy, the maximum flux of IAA that is achieved is 2.18 mmol/(gDW h). IAA overproduction is achieved by deleting 13 genes individually in the model, that is, one at a time as it is a deletion strategy. The highest production of IAA is achieved when the *trp*A (BSU13350) and or *spl*B (BSU13930) genes are deleted. The first of these elements encodes a chain tryptophan synthase enzyme and, in the model, this enzyme participates in the formation of Trp from L-serine, or vice versa, due to the fact that it is bidirectionally annotated. Therefore, when Trp is found in the medium, the formation of L-serine from Trp is favored, deviating the production of IAA. The *spl*B gene according to the SubtiWiki database is associated with sporulation processes [43], but in the model, it is found as a gene associated with the enzyme that catalyzes the interconversion reaction between pyruvate and iso-chorismate metabolites related to the synthesis of chorismate. Out of the remaining candidate genes to be eliminated, there are seven elements that participate in the synthesis of teichoic acid and although the elimination strategy considers them non-essential, these are reported as essential genes for growth in LB medium in the SubtiWiki database, so they should not be removed.

#### 3.4.4. Phenotype Phase Plane Analysis 

**Figure 8:**
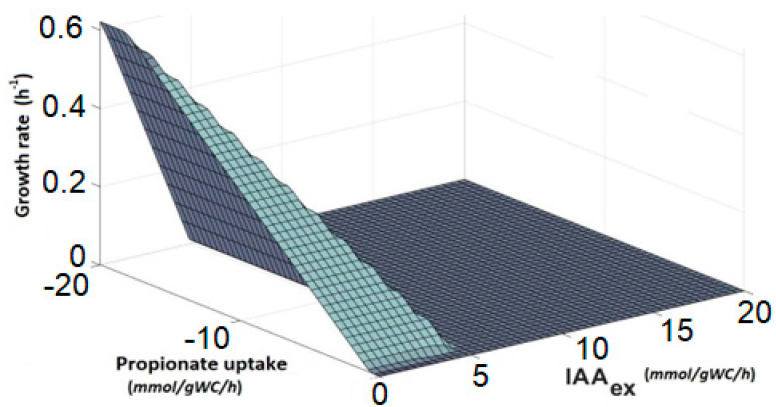
Phenotype Phase Plane analysis of propionate metabolism as a function of biomass and IAA production.

The modified *i*BSU1147 genomic-scale metabolic model generates a Phenotype Phase Plane analysis (PhPP) which describes the metabolic transition of *B. subtilis* depending on the availability of sodium propionate and IAA production. The three-dimensional response surface corresponds to the growth rate variation for each pair of rates of propionate uptake and IAA formation in the x–y plane, see Figure 8. All flux distributions in the metabolism of *B. subtilis* are limited to a solution region where each distribution reflects a feasible metabolic flux [44]. In this particular case, we were interested in the relationship between the *q_s_* of the propionate and the growth rate during the pseudo-stationary phase and during the formation of the product. As shown in Figure 8, low values of propionate uptake are not enough to complete the metabolic functions required for cell maintenance and production of IAA. As this rate value increases, the growth rate begins to increase linearly. 

#### 3.4.5. Bilevel Optimization

The bilevel optimization algorithm, OptKnock, predicts those reactions that can be deleted in order to increase the IAA production maintaining the maximum level of growth rate. Table 2 contains the set of reactions that can be deleted and slightly increases the IAA production maintaining the optimal growth rate. The strategies using propionate predict higher productivity compared to those predicted when glucose is consumed. 

The deletion strategies from Table 2 consider the deletion of the malate dehydrogenase, *mdh* and the multimeric enzyme alpha-ketoglutarate dehydrogenase codified by the genes *odhA*, *odhB*, *pdhD* [45,46]. The set of genes is not considered essential in *B. subtilis*.

Other strategies are the deletion of the ribulose-5′phosphate epimerase and the fructokinase. These two reactions are associated with the production of intermediates of the shikimate pathway, essential to produce Trp. The deletion of the gene *pfkA* conducts to the accumulation of fructose-6-phosphate. 

**Figure 9:**
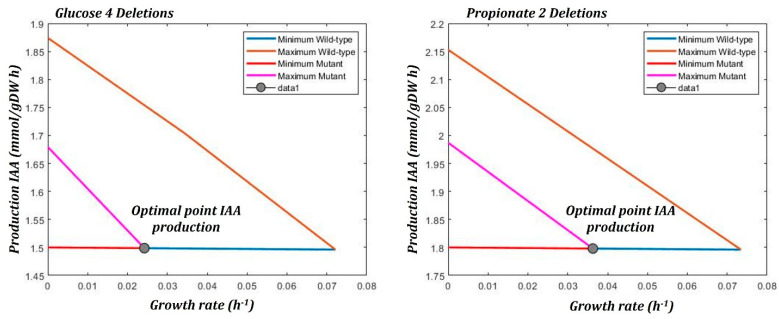
Graphs obtained from the simulation with OptKnock. The intersection point corresponds to the optimal solution.

Figure 9 presents the solutions found from OptKnock. The lines are the limits when maximizing and minimizing the IAA production in the mutant and wild type network with their respective biomass yield, this is, when the IAA production is maximized or minimized the biomass production is calculated. It can be seen that in either network, wild type and mutant, the linear relationship has a similar dependency. The optimal point lies on the horizontal edge of the optimization front and indicates that the maximization of biomass and IAA production are coupled in the scenarios when two and four deletions are made, see Table 2.

## 4. Discussion

IAA production in *B. subtills* is Trp dependent. Despite this dependency, the productivity and yield variates according to the carbon sources. The cultivation under controlled conditions of pH and temperature demonstrates that propionate improves the metabolism for IAA production, (see Table 1). According to the flux distribution, the incorporation of propionate through the methylcitric acid pathway modifies the activity in the TCA providing a metabolic precursor for IAA. The product formation is favored during the stationary growth phase, where the characteristic is a low value in the growth rate. Under this physiological condition, the IAA production is favored, the experimental observations and the computational prediction support this conclusion. With robustness analyses, the effect related to the increment of IAA production vs. the decrease in the specific growth rate can be associated to the nature of secondary metabolism that produces the indole derivatives, such as indole-3-acetamide and IAA. Another parameter that was computationally evaluated is the impact of oxygen consumption, as the presence of this metabolite with different O_2_ radicals negatively affecting the synthesis of IAA because Trp; these oxidations can result in the formation of a mixture of several products and prevent the conservation of the indole ring and therefore, cannot participate in the synthesis of IAA [47]. In addition, O_2_ is essential to carry out oxidative phosphorylation, it can be seen that basal O_2_ consumption must be present to promote sodium propionate metabolism. Hu et al. [48] and Junne et al. [49] used batch culture strategies to observe the attribution of oxygen consumption in the formation of a determined product and how its availability could constitute a limiting factor. In both studies, it can be seen that an increment in the consumption of O_2_ also conducts an increment in substrate consumption, so the availability of this gas is essential for the optimal use of sodium propionate and the formation of IAA. The evolution of the different phenotypic phase planes shows which changes are manifested in the cell as a function of the environmental variables such as propionate uptake and IAA production. Therefore, this type of analysis allows us to see how different external perturbations significantly impact the phenotype of a strain, and therefore, in the formation of a product of interest. Based on this observation, an excess in propionate uptake is not a variable that impacts the IAA productions. Therefore, the modulation in propionate uptake and growth rate can result in the optimal production of IAA. 

To have a dimension of the potential of IAA production using propionate and minimal media we can compare our results with the previously reported, see Table 3. 

In metabolic engineering, one of the strategies to improve product formation is the deletion of selected genes. Single-gene deletion analysis suggests a strategy based on the elimination of reactions for the overproduction of specific compounds, with this strategy it was possible to identify 13 genes, from this list the most relevant are the genes related to de novo synthesis of tryptophan, see Appendix B. On the other hand, we identified that 249 genes are essential for IAA production (see Supplementary material 8). Based on multilevel optimization, other deletion strategies were found to increase IAA production; nevertheless, in this case, it is important to be cautious as there is a strong objective coupling between the maximization of IAA production and the maximization of cell production.

Several parameters increase the production of IAA, among them are, greater availability of oxygen, the consumption of Trp and the carbon source, and the optimization of the production pathway of this metabolite. Considering the results presented, possible strategies for the overproduction of IAA might be viable using *Bacillus subtilis* [51]. The insertion of genes such as *iaa*M (tryptophan monooxygenase) with greater efficiency to favor the formation of IAA from IAM, as it was proposed by Defez et al. [52]. Another strategy is the modification in regulatory pathways, for instance, the engineering of transcription factors and the dynamic regulation of the *trp*EDCBA operon and its control elements such as the *trp*R repressor protein, as well as the engineering of amino acid transport systems inside the cell [53]. On the one hand, strategies that increase the intracellular concentration of essential metabolites for Trp synthesis pathways such as PEP and E4P are added to pathway control. According to the results obtained through OptKnock, genes such as malate dehydrogenase (*mdh*) or pyruvate kinase (*pyk*) can be eliminated and in this way avoid the consumption of PEP [54].

## Figures and Tables

**Table 1:** Kinetics parameters of *Bacillus subtilis* cultivation in a 3 L bioreactor comparing two carbon sources, glucose and propionate.

Parameter	Glucose	Propionate
*μ*_exp_	0.15 ± 0.06	0.14 ± 0.02
*μ*_stat_	0.07 ± 0.12	0.06 ± 0.04
*q*_s,exp_	2.15 ± 0.08	4.18 ± 0.04
*q*_s,stat_	1.2 ± 0.03	2.85 ± 0.01
*q*_s,Trp_	1.5 ± 0.05	1.8 ± 0.02
*q*_p,IAA_	1.33 ± 0.02	1.92 ± 0.07
Y_x/s_	0.324 ± 0.05	0.219 ± 0.08
Y_x/(s+Trp)_	0.134 ± 0.11	0.094 ± 0.06
Y_p/(s+Trp)_	0.446 ± 0.03	0.524 ± 0.04
Y_p/Trp_	0.761 ± 0.04	0.915 ± 0.03

Specific growth rates (*μ*) have units of h^−1^, specific rates (*q*) have units of mmol/(gDW h). Yields have units of g/g. Subindexes are, substrate (s) and product IAA (p).

**Table 2:** Sets of candidate reactions to be deleted with potential increase in the production of IAA while maintaining optimal biomass production.

GLUCOSE			Max ⱱ_bio_	
No. Del	Knockouts	Enzyme	Biomass (h^−1^)	IAA (mmol/gDW h)
0	“Wild type”	*-------*	0.1	1.496
2	1. FDP <=> DHAP + G3P 2. MAL <=> FUM + H_2_O	Fructose bisphosphate aldolase (*fbaA*) Fumarase (*citG*)	0.07	1.4992
3	1. 2PG <=> H_2_O + PEP 2. MAL <=> FUM + H_2_O 3. G6P <=> F6P	Enolase (*eno*) Fumarase (*citG*) Glucose-6-phosphate isomerase (*pgi*)	0.04	1.51
4	1. MAL + NAD <=> OAA + H + NADH 2. RU5P <=> XU5P 3. AKG + NAD + CoA<=> SUC-CoA + CO_2_ + NADH 4. F6P + ATP -> FDP + ADP	Malate dehydrogenase (*mdh*) Ribulose-5-phosphate epimerase (*rpe*) α-Ketoglutarate dehydrogenase (*odhB*) Phosphofructokinase (*pfkA*)	0.04	1.61
**PROPIONATE**			**Max ⱱ_bio_**	
**No. Del**	**Knockouts**	**Enzyme**	**Biomass (h^−1^)**	**IAA (mmol/gDW h)**
0	“Wild type”	*-------*	0.1	1.796
2	1. MAL + NAD <=> OAA + H + NADH 2. SUC-CoA + GDP + HPO_4_ <=> SUC + GTP + CoA	Malate dehydrogenase (*mdh*) Succinyl-CoA synthetase (*sucCD*)	0.06	1.798
3	1. MAL + NAD <=> OAA + H + NADH 2. 2PG <=> H_2_O + PEP 3. AKG + NAD + CoA<=> SUC-CoA + CO_2_ + NADH	Malate dehydrogenase (*mdh*) Enolase (*eno*) α-Ketoglutarate dehydrogenase (*odhB*)	0.03	1.798
5	1. AKG + ALA <=> GLU + PYR 2. MAL + NAD <=> OAA + H + NADH 3. 2PG <=> H_2_O + PEP 4. AKG + VAL <=> GLU + 3M-2-OB 5. AKG + NAD + CoA<=> SUC-CoA + CO_2_ + NADH	Transaminase (*alaT*) Malate dehydrogenase (*mdh*) Enolase (*eno*) Transaminase (*ywaA*) α-Ketoglutarate dehydrogenase (*odhB*)	0.06	1.798

**Table 3:** Comparative table of IAA production by *Bacillus subtilis* in different culture media.

Authors	Concentration (g/L)	Medium
Araujo et al., 2005 [10]	0.17	Potato-dextrose (PDA)
Zaidi et al., 2006 [11]	0.055	M9 + Glucose
Sivasakthi et al., 2013 [50]	0.025	Agar Nutriente
Alfonso et al., 2021 [30]	0.086	M9 + Propionate
This work	0.31	M9 + Propionate

## Data Availability

Not applicable.

## Supplementary Materials

The following supporting information can be downloaded at: https://www.mdpi.com/article/10.3390/microorganisms10122352/s1. These are the supplementary materials that are included in the manuscript: Supplementary material 1—Excel file that includes the information on the growth kinetics in the flask experiments (“biological replicates”). Technical replicates on HPLC measurements for substrate consumption and IAA production, as well as statistical analysis. Supplementary material 2—Excel file that include Curve Pattern Standard. Supplementary material 3—Flux Balance Analysis Programming Code. Supplementary material 4—OptKnock Programming Code. =Supplementary material 5—Single gene deletion. Robustness Analysis (Programming code). Supplementary material 6—Genome scale metabolic models *i*BSU1147+1 [.mat]. Supplementary material 7—Genome scale metabolic models *i*BSU1147+1 [.xls]. Supplementary material 8—Excel file that include Single Gene Deletion analysis.

## Appendix A

**Table A1:** List of reactions used for the flux analysis in the metabolic pathway.

glucose –> or propionate –>
g6p <=> f6p
atp + f6p <=> adp + f1,6bf
f1,6bf <=> g3p + dhp
g3p + nad + pi <=> 1,3dpg + h + nadh
atp + h + 3pg <=> adp + 1,3dpg
2pg <=> 3pg
3pg <=> h2o + pep
adp + pep <=> atp + pyr
pyr + Coa + nad <=> acoa + nadh + co_2
ACoa + h_2o + oaa <=> h + coa + cit
cit <=> acon-C + h_2o
acon-C + h_2o <=> icit
icit + nadp <=> akg + co_2 + nadph
akg + Coa + nadh –> co_2 + nadh + succoa
atp + Coa + succ <=> adp + pi + succoa
q8 + succ –> fum + q8h2
fum + h_2o <=> mal
mal + nad => h + nadh + oaa
atp + oaa –> adp + co_2 + pep
atp +pyr <=> adp + p_i + h + oaa
mal-L + nad –> co_2 + nadh + pyr
mal-L + nadp –> co_2 + nadph + pyr
g6p + nadp <=> 6pgl + h + nadph
6pgc + nadp –> co_2 + nadph + ru5p
ru5p <==> xu5p
r5p <=> ru5p
e4p + xu5p <=> f6p + g3p
g3p + sh7p <=> f6p + e4p
ser + igp <=> g3p + h_2o + trp
tryptophan –>
trp <=> iam
h_2o + iam <=> nh_4 + iaa

## Appendix B

**Table A2:** List of genes from the Single Gene Deletion analysis.

Locus	Flux	Protein
BSU13350	2.18	tryptophan synthase alpha chain
BSU13930	2.18	undespore photoproduct lyase
BSU21920	2.15	processive diacylglycerol glucosyltransferase
BSU35760	1.77	glycerol phosphate glycerophosphotransferase
BSU35660	1.77	UDP-N-acetylglucosamine 2-epimerase
BSU35700	1.77	ABC transporter
BSU35710	1.77	ABC transporter
BSU3572	1.75	glycerol-phosphotransferase
BSU35530	1.75	GlcNAc-1-phosphate transferase
BSU35750	1.74	UDP-N-acetyl-D-mannosamine transferase
BSU35730	1.72	polyglycerol phosphate GlcNAc-1-phosphate transferase
BSU35750	1.74	UDP-N-acetyl-D-mannosamine transferase
BSU35730	1.72	polyglycerol phosphate glucosyltransferase
